# Contextual variables affect peak running performance in elite soccer players: A brief report

**DOI:** 10.3389/fspor.2022.966146

**Published:** 2022-09-16

**Authors:** Diêgo Augusto, João Brito, Rodrigo Aquino, Dailson Paulucio, Pedro Figueiredo, Bruno Luiz Souza Bedo, Deborah Touguinhó, Fabrício Vasconcellos

**Affiliations:** ^1^Laboratory of Soccer Studies (LABESFUT), Post-graduate Program in Exercise and Sport Sciences, Institute of Physical Education and Sports, State University of Rio de Janeiro, Rio de Janeiro, Brazil; ^2^Portugal Football School, Portuguese Football Federation, Oeiras, Portugal; ^3^Department of Sports, Center for Physical Education and Sports, Federal University of Espírito Santo, Vitória, Espirito Santo, Brazil; ^4^Biometrics Laboratory (LADEBIO), Physical Education Post-graduate Program, School of Physical Education and Sports (PPGEF/UFRJ), Federal University of Rio de Janeiro, Rio de Janeiro, Brazil; ^5^Physical Education Department, College of Education, United Arab Emirates University, Al Ain, Abu Dhabi, United Arab Emirates; ^6^Research Center in Sports Sciences, Health Sciences and Human Development, CIDESD, Vila Real, Portugal; ^7^School of Physical Education and Sport, University of São Paulo, São Paulo, Brazil

**Keywords:** match analysis, GPS, tracking, team sports, worst-case scenario

## Abstract

The current brief research report aimed to investigate the influence of contextual variables on peak running performance in male elite soccer players. We analyzed 29 matches of an elite soccer team during the Brazilian Serie A 2019. Twenty players were tracked using GPS units. Peak physical performance was determined using moving average running values with different time windows (1, 3, and 5-min periods). The variables analyzed were total distance covered, total distance covered in high-intensity running (≥19.8 km·h^−1^), and the distance in accelerations (≥2 m·s^−2^) and decelerations ( ≤-2 m·s^−2^). Four contextual variables were considered: 1) positional status; 2) match location; 3) match outcome; and 4) match status. Central defenders showed a lower 1-min peak total distance in relation to all other positions (*p* = 0.001–0.03). Peak physical performance was higher in away matches for high-intensity running, acceleration, and deceleration (*p* = 0.01–0.03). In matches that ended in losses, peak values for high-intensity running and acceleration were higher compared to draws and wins (*p* = 0.01–0.04). Regarding the match status, higher values were observed in draws than wins and losses (*p* = 0.01). Peak running performance vary according to contextual variables of the match in male elite soccer players. Positional differences were found for peak periods, and physical performance was higher in away matches.

## Introduction

The use of absolute values of external load obtained during a soccer match has been applied by technical staff for training prescription (Bradley et al., 2010; Barnes et al., 2014; Aquino et al., 2021). However, the scientific literature has reported that using values referring to absolute demands can underestimate the match requirements and, therefore, not transfer the actual value of running to training (Fransson et al., 2017; Casamichana et al., 2019). In this sense, the peak physical performance can add value for a better understanding of the demands of match play (Novak et al., 2021; Rico-González et al., 2021).

The utilization the peak periods, has been recommended primarily in moving averages of 1, 3, 5, and 10 min (Martín-García et al., 2018). Different studies have indicated that the moving average technique is considered more adequate when compared to fixed intervals (Casamichana et al., 2019; Oliva-Lozano et al., 2020). In addition, different running metrics have been used, total distance, high intensity running, sprinting, accelerations and decelerations (Whitehead et al., 2018). These analyzes of peak periods help in the organization of training microcycles, in addition, athletes analyze in a sport that can meet the real requirements of the match (Oliva-Lozano et al., 2022).

Peak periods of physical demand have been studied according to positional and contextual factors. For instance, the position, match half, location, and outcome impacted players' peak of physical demands (Oliva-Lozano et al., 2020). Furthermore, peak periods for high-intensity running can vary by up to 38%, and sprint running by up to 75% during the competitive season (Novak et al., 2021). Other contextual factors, such as the style of play and cultural factors of each soccer league, can generate variations in the values of physical demand in soccer matches (Dellal et al., 2011).

Notably, contextual variables can influence several match outcomes (Aquino et al., 2020; Augusto et al., 2021; Gonçalves et al., 2021). For example, total distance and high-intensity running in home matches were higher than in away matches (Aquino et al., 2020). In matches won, the distance covered per minutes at high intensity increased compared to ties and losses (Gonçalves et al., 2021). Regarding the opponent's level, studies have indicated that playing against teams considered stronger causes higher running values (Aquino et al., 2017, 2020). However, few studies have investigated the effects of contextual variables in the most demanding periods in elite soccer players to the best of our knowledge (Fransson et al., 2017; Casamichana et al., 2019). A better understanding of the influence of the context on peak performance is paramount for a better prescription of training sessions (Wass et al., 2020; Ju et al., 2021). Therefore, the present study aimed to investigate the influence of contextual variables on peak physical performance in elite male soccer players.

## Methods

The present observational brief research report analyzed 29 matches from one team competing in the Brazilian Serie A 2019. The study was conducted following the Declaration of Helsinki and was approved by the local university ethics committee (3.712.816).

### Participants

Data were analyzed exclusively for 20 elite male soccer outfield players (25.7 ± 4.4 years; 180.1 ± 6.1 cm; 75.4 ± 7.8 kg) who played the full 29 matches (minimum 90 min played per match). There were 4 Central Defenders, 4 Fullbacks, 7 Midfielders, and 5 Forwards. Goalkeepers were excluded from the sample. In total, 175 player observations were considered. The tactical systems used in the season were: 1–4–4–2, 1–4–3–3 and 1–3–5–2.

### Procedures

Each player wore the same 10-Hz GPS unit (Viper pod, STATSports, Belfast, United Kingdom) throughout the matches (Jennings et al., 2010; Beato et al., 2018). Following the matches, data were downloaded using corporative software, and raw data were exported for further analysis in the Matlab environment (The MathWorks Inc., Natick, USA). Geographic coordinates were transformed to cartesian coordinates (*x, y*) and smoothed with a Butterworth digital filter (third-order; cut-off frequency: 0.3 Hz). After that the physical variables were calculated.

### Independent variables

The following metrics were gathered: total distance (TD) and total distance covered in high-intensity running (HIR, ≥19.8 km·h^−1^), acceleration (≥2 m·s^−2^), and deceleration (≤-2 m·s^−2^). The moving average method was applied to calculate the worst-case peak physical performance during the match (Whitehead et al., 2018). The average of the 1-, 3-, and 5-min periods were calculated and normalized by the time in minutes, so that all metrics were presented in m/min. The 1-min peak identified from the averages determined as each peak (maximum) value was selected for statistical analysis.

### Contextual variables

Four contextual variables were considered: (i) Positional status was defined from the calculation of the average position of each player using Cartesian coordinates. The position in each game was considered, that is, the same player can change position in the analysis according to the game (Central Defender = 49; Fullback = 46; Midfielder = 45; Forward = 45, observations); (ii) Match location (Home = 67; Away = 108, observations); (iii) Match outcome: Defined as the end result of the match (Win = 60; Draw = 57; Losses = 58, observations); (iv) Match status: Defined as the momentary result during the match (Winning = 730; Drawing = 1,458; Losing = 557, minutes).

### Statistical analysis

Data were described as mean and 95% confidence interval (CI). The Kolmogorov-Smirnov normality test was performed. Variables that did not show normality were corrected and log-transformed, after which all variables were considered parametric by the same normality test. Separate linear mixed models were performed to compare (fixed effects) positional status, match location, match outcome, and the match status, and to perform within-subject analysis, the ‘player ID' was included as a random effect. The best-fitting covariance structure minimized Hurvich and Tsai's criterion value (Hurvich and Tsai, 1989). In addition, multiple comparisons were adjusted by the Bonferroni method. The *t*-statistical values of the mixed models were converted into correlations, and their effect size (ES) interpreted as follows: trivial (ES < 0.1), small (ES = 0.1–0.3), moderate (ES = 0.3–0.5), large (ES = 0.5–0.7), very large (ES = 0.7–0.9), and almost perfect (ES > 0.9) (Hopkins et al., 2009). The significance value adopted was *p* ≤ 0.05.

## Results

The values observed during the matches for the different variables analyzed were: peak total distance (1-min [182±21 m/min] 3-min [138±15 m/min], 5-min [126±13 m/min]), peak high intensity running (1-min [18±11 m/min], 3-min [11±7 m/min], 5-min [9±3 m/min]), acceleration (1-min [8±4 m/min], 3-min [5±2 m/min], 5-min [4±1 m/min]), and deceleration (1-min [9±5 m/min], 3-min [6±3 m/min], 5-min [5±2 m/min]).

**Figure 1:**
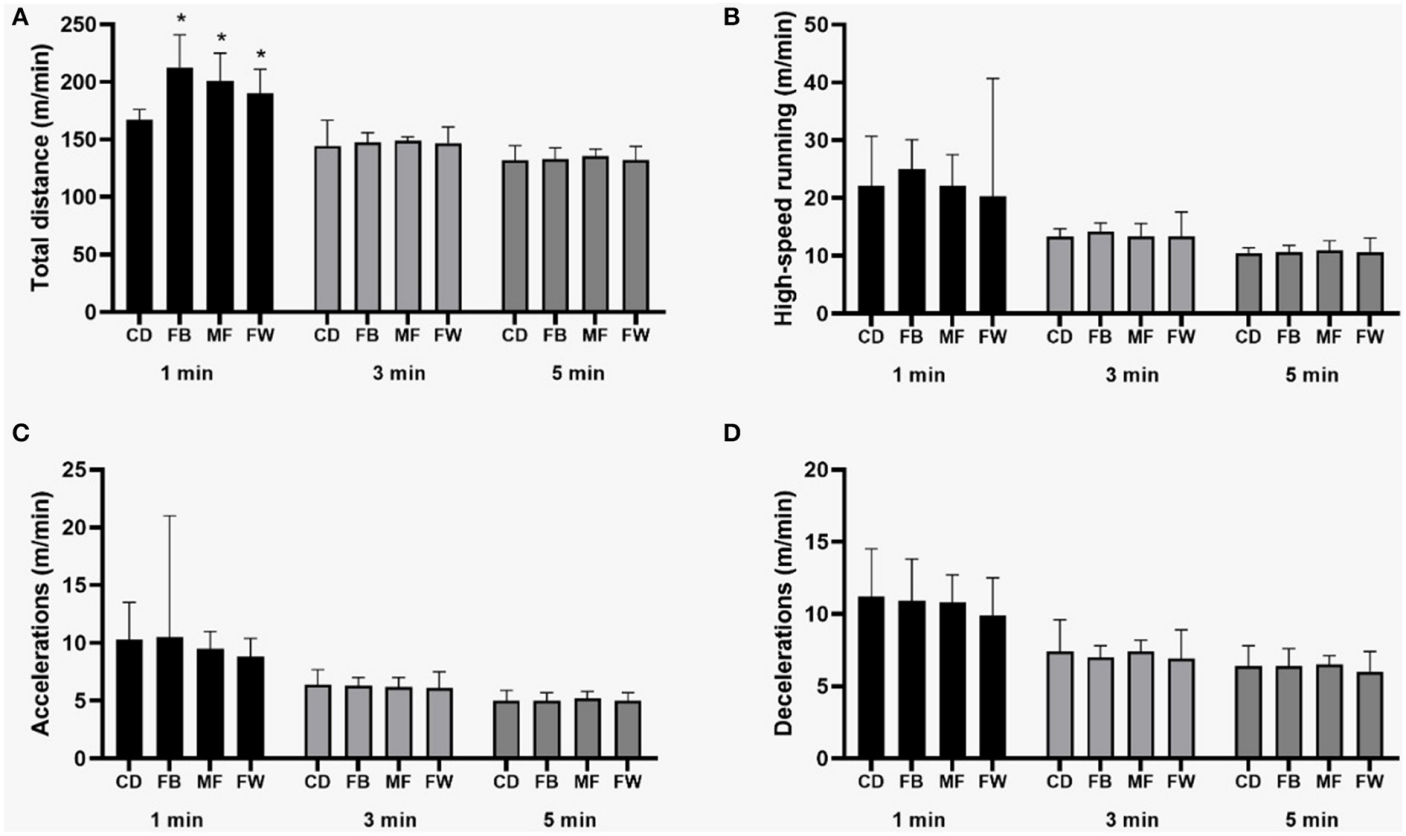
Mean values and 95% CI of physical performance peaks in different metrics and periods according to the positional status. * >Central Defender. **(A)** Total distance; **(B)** High-speed running; **(C)** Accelerations; **(D)** Decelerations. CD; Central Defender, FB; Fullback, MF; Midfielder, FW; Forward.

**Figure 2:**
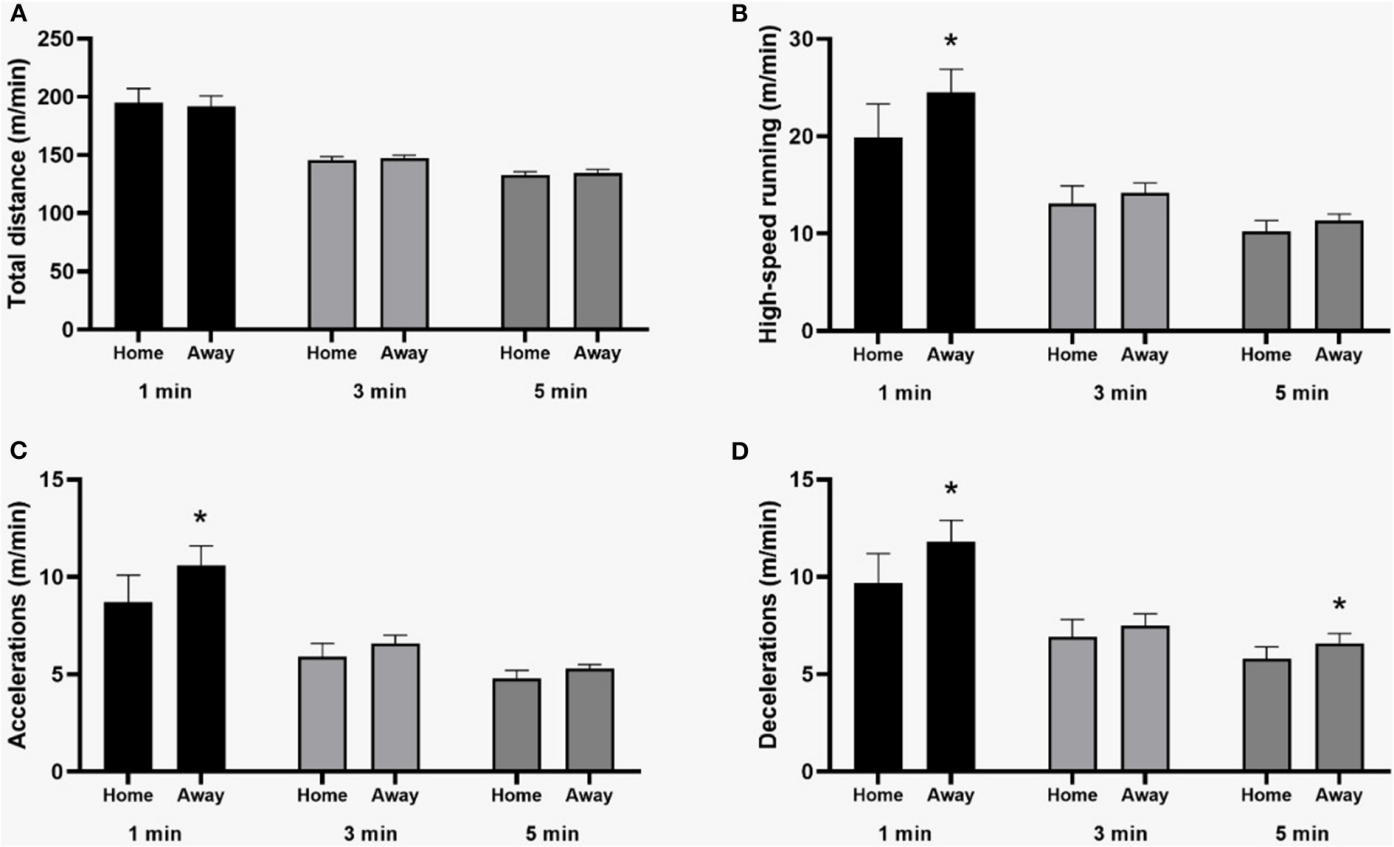
Mean values and 95% CI of physical performance peaks in different metrics and periods according to the match location. *Away > Home. **(A)** Total distance; **(B)** High-speed running; **(C)** Accelerations; **(D)** Decelerations.

In relation to the positional status, forwards (*p* = 0.03; ES = 0.99), fullbacks (*p* = 0.01; ES = 0.99), and midfielders (*p* = 0.001; ES = 0.99) covered a greater peak total distance in the 1-min moving average when compared to central defenders (Figure 1). Regarding the location of the match, the peak of high intensity running (*p* = 0.03; ES = 0.19) and acceleration (*p* = 0.03; ES = 0.31) were higher in away matches in the 1-min period. Similarly, the distance in deceleration was higher in away matches in periods of 1- (*p* = 0.05; ES = 0.16) and 5-min (*p* = 0.03; ES = 0.37) (Figure 2).

**Figure 3:**
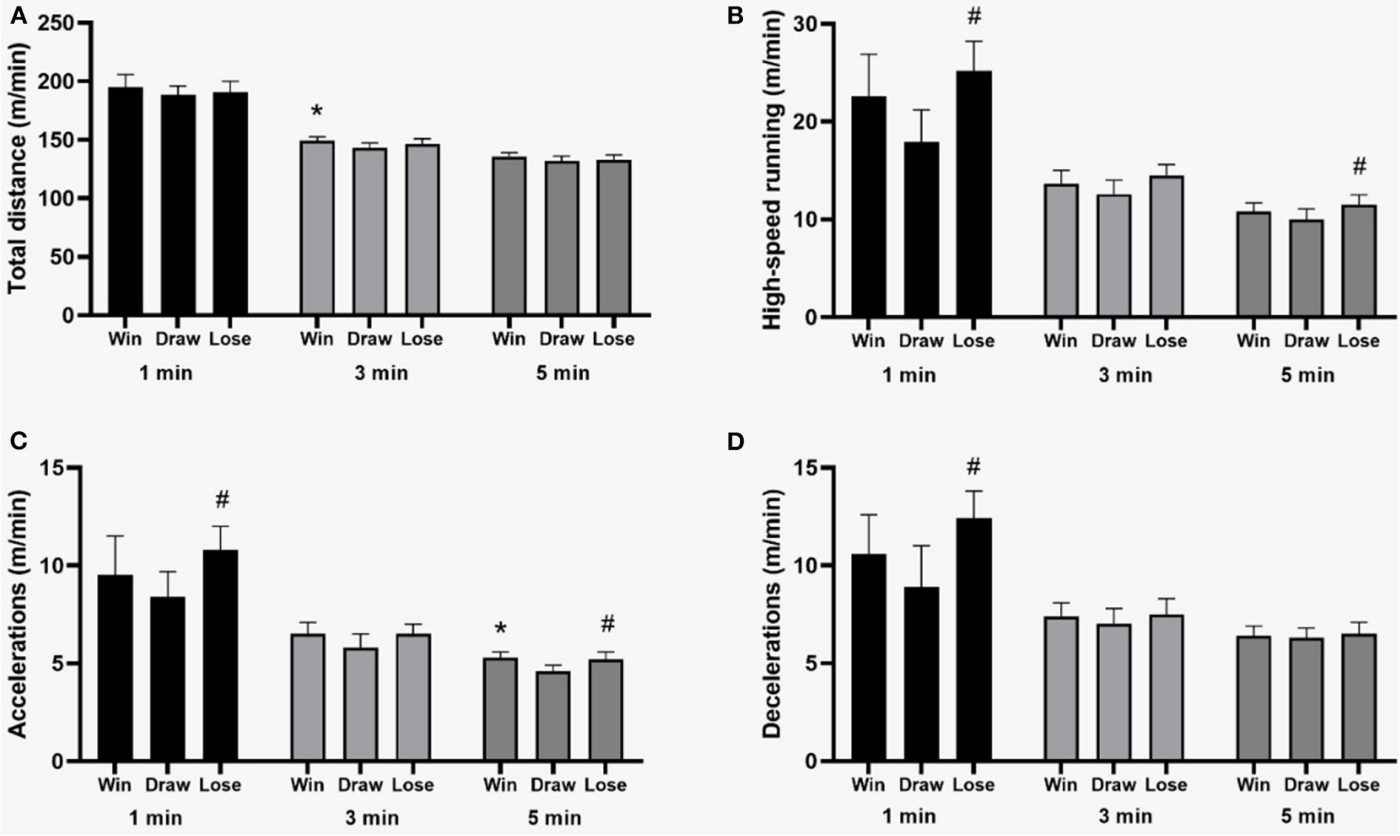
Mean values and 95% CI of physical performance peaks in different metrics and periods according to the outcome match. *Win > Draw; ^#^Lose > Draw. **(A)** Total distance; **(B)** High-speed running; **(C)** Accelerations; **(D)** Decelerations.

When the analyzed team lost the match, there was a higher peak of distance covered in high-intensity running in the 1- and 5-min periods compared to the situation of draws (*p* = 0.01; ES = 0.38 and *p* = 0.02; ES = 0.27, respectively) (Figure 3). In losses, there was also a higher peak distance in acceleration (*p* = 0.04; ES = 0.37) and deceleration (*p* = 0.01; ES = 0.40) in the 1-min moving average compared to draws. In addition, when the team won the match, higher peak of total distance covered was identified compared to draws in 3-min periods (*p* = 0.03; ES = 0.39).

**Figure 4:**
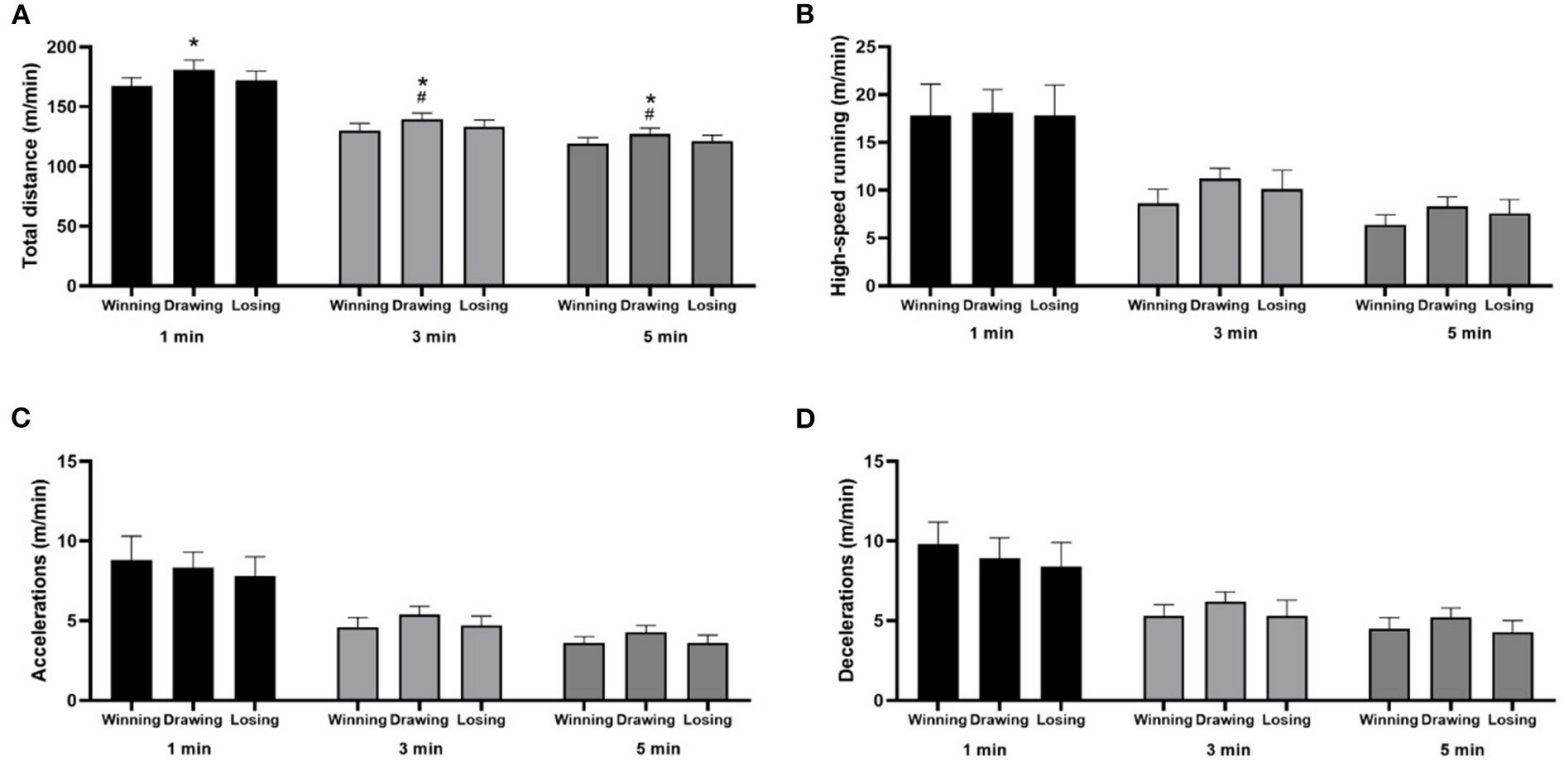
Mean values and 95% CI of physical performance peaks in different metrics and periods according to the match status. *Drawing > Winning; ^#^Drawing > Losing. **(A)** Total distance; **(B)** High-speed running; **(C)** Accelerations; **(D)** Decelerations.

Players covered a higher peak of total distance in 1-, 3-, and 5-min periods when winning the match (*p* < 0.01; ES = 0.55–0.84) compared to draws and losses. Also, higher peak distance in 3- (*p* = 0.01; ES = 0.64) and 5-min (*p* = 0.01; ES = 0.52) periods was observed when drawing than loosing (Figure 4).

## Discussion

The present brief research report aimed to investigate the effects of contextual variables on peak physical performance in elite male soccer players in Brazil. The main findings indicate that: i) positional differences were found for peak periods; ii) in away matches, physical performance during peak values was higher, and iii) in draws, the peak period outcomes were lower than during wins and losses.

In the comparisons by field positions, defenders showed lower values when compared to all other positions. These results are consistent with data found by others (Martín-García et al., 2018; Casamichana et al., 2019; Oliva-Lozano et al., 2020). The lower peak physical performance values for defenders may result from the situational demands of the position. For instance, the physical tasks of central defenders are primarily recovery runs, compaction, and coverage (Bradley and Ade, 2018; Ju et al., 2021). Then, from a physical perspective, defenders might present lower physical demands for the general values of a full match and the peak values (Di Mascio and Bradley, 2013; Bush et al., 2015; Barrera et al., 2021).

Other factors, such as the influence of playing at home, have also drawn the attention of researchers from different countries (Lago-Peñas and Lago-Ballesteros, 2011; Aquino et al., 2020; Barrera et al., 2021). In the present brief research report, it was observed that in away matches, the 1-min period peak values of distance in high intensity and acceleration and 1- and 5-min periods in deceleration were higher when compared to home matches. These results agree with the results found by Oliva-Lozano et al. (2020), that also observed higher values in away matches (Oliva-Lozano et al., 2020). However, compared with other results found in studies that followed absolute values of matches in Brazil and in Portugal, there is a divergence (Aquino et al., 2017, 2020; Barrera et al., 2021), but it is worth mentioning that these studies were carried out at the sub-elite level and did not analyze the peak periods. Furthermore, Castellano et al. (2011) found no difference for distances covered at different intensities between home and away matches in Spanish Premier League (Castellano et al., 2011).

Regarding the match outcome, higher values of total peak distance covered in 1-min periods were observed in matches that ended with wins, and higher values of running in high intensity, acceleration, and deceleration in losses. When compared to draws, soccer players in Spain showed higher values of peak periods in wins than in draws and losses (Oliva-Lozano et al., 2020). Similarly to Oliva-Lozano et al. (2020) findings, the results of the present brief research report might be related to the fact that there is a need to equalize the score in defeat situations, resulting in a more significant occurrence of intense actions (Oliva-Lozano et al., 2021).

Moreover, when the match status during the match was analyzed, it was found that the highest peak values for total distance covered 1-, 3-, and 5-min periods in situations of equality in the score when compared with winning and losing. To the best of our knowledge, this is the first study to analyze the influence of the match status on the peak physical performance of elite soccer players in Brazil. A study from Spain found that players covered greater distances in all intensity range categories when losing (Castellano et al., 2011). This contextual variable has been little explored and limits the discussion and comparisons with previous studies. However, the results presented can be explained by the fact that, despite the application of tactical principles being the main factor for the offensive and defensive organization of the team. The situation of equal results can evidence the players' search to “unbalance” the opponents, performing a greater number of physical actions. For example, physical actions can be performed in situations such as Break into Box, Over/Underlap and Run with Ball (Ju et al., 2021). This result can motivate the performance of a more significant number of physical actions (Castellano et al., 2011; Buchheit et al., 2018; Klemp et al., 2021).

Coaches and sports scientists can use the results of the current brief research report to improve training tasks in return-to-play and post-injury conditioning sessions. Although peak performance moments vary due to the context, this should be achieved in training planning to prepare players for the practical match demands. In addition, this is the first study conducted with male elite soccer players in Brazil reporting the peak values of physical performance during official matches. Therefore, future research should analyze the peak moments in the Brazilian scenario involving different teams and competitions for a better understanding. In addition, to understand how the moments of running peaks occur in weekly microcyles.

## Data availability statement

The datasets generated for this study are available on request to the corresponding author.

## Ethics statement

The studies involving human participants were reviewed and approved by State University Rio de Janeiro. The patients/participants provided their written informed consent to participate in this study.

## Author contributions

DA: conceptualization, data curation, project administration, formal analysis, investigation, methodology, visualization, and writing—review and editing. JB: data curation, formal analysis, methodology, visualization, and writing—review and editing. RA: conceptualization, data curation, methodology, and writing—review and editing. PF: formal analysis, methodology, and data curation. BB and DP: data curation and methodology. DT: data curation and investigation. FV: conceptualization, methodology, project administration, supervision, and visualization. All authors contributed to the article and approved the submitted version.

## Funding

This research was partially supported by grants from the Carlos Chagas Filho Foundation for the Research Support in Rio de Janeiro State and Brazilian Council for the Technological and Scientific Development and was financed in part by the Coordenação de Aperfeiçoamento Pessoal de Nivel Superior-Brasil (CAPES)-Finance Code 001.

## Conflict of interest

The authors declare that the research was conducted in the absence of any commercial or financial relationships that could be construed as a potential conflict of interest.

## Publisher's note

All claims expressed in this article are solely those of the authors and do not necessarily represent those of their affiliated organizations, or those of the publisher, the editors and the reviewers. Any product that may be evaluated in this article, or claim that may be made by its manufacturer, is not guaranteed or endorsed by the publisher.
